# Reversible predominant thyrotropin axis dysfunction with meningitis in suspected lymphocytic hypophysitis

**DOI:** 10.1210/jcemcr/luag221

**Published:** 2026-08-12

**Authors:** Kohei Iinuma, Ryota Amano, Katsumi Taki

**Affiliations:** Department of Endocrinology and Metabolism, Fujiyoshida Municipal Medical Center, Fujiyoshida City, Yamanashi 403-0032, Japan; Department of Neurology, Fujiyoshida Municipal Medical Center, Fujiyoshida City, Yamanashi 403-0032, Japan; Department of Endocrinology and Metabolism, Fujiyoshida Municipal Medical Center, Fujiyoshida City, Yamanashi 403-0032, Japan

**Keywords:** lymphocytic hypophysitis, meningitis, predominant thyrotropin axis dysfunction, central hypothyroidism, pituitary inflammation, reversible hypopituitarism

## Abstract

Lymphocytic hypophysitis (LYH), attributed to autoimmune mechanisms, typically causes multiple anterior pituitary hormone deficiencies, with corticotroph dysfunction occurring earliest; predominant thyrotroph dysfunction is uncommon. A 65-year-old woman presented with headache, fever, and diplopia. Laboratory tests revealed marked systemic inflammation and cerebrospinal fluid (CSF) pleocytosis. Pituitary magnetic resonance imaging (MRI) demonstrated diffuse enlargement and homogeneous contrast enhancement of the pituitary gland and stalk. Endocrinological evaluation showed paradoxically decreased thyroid-stimulating hormone (TSH) despite low thyroid hormone levels, contrasting with elevated adrenocorticotropic hormone (ACTH) levels. Endocrinological stimulation tests revealed a markedly blunted TSH response, with preserved gonadotropin and growth hormone responses, indicating predominant thyrotroph dysfunction despite preserved corticotroph function. Glucocorticoid therapy resulted in prompt improvement of clinical, laboratory, and MRI findings, with complete recovery of anterior pituitary functions. A presumptive diagnosis of LYH with reversible predominant thyrotroph dysfunction and meningitis secondary to direct extension of pituitary inflammation was established. This case demonstrates that LYH can present with atypical anterior pituitary hormone dysfunction, may be complicated by aseptic meningitis, and requires prompt glucocorticoid therapy for favorable outcomes.

## Introduction

Hypophysitis is a rare inflammatory disorder of the pituitary gland and stalk, typically presenting with headache and pituitary hormone dysfunction [1, 2]. Although primarily attributed to autoimmune mechanisms involving lymphocytic infiltration, hypophysitis has a broad etiology [1-3]. Pituitary biopsy is useful for excluding other etiologies; however, no established diagnostic criteria exist in adults, and a presumptive diagnosis is often made without biopsy [1, 3].

In lymphocytic hypophysitis (LYH), anterior pituitary deficiencies typically occur in the following order: corticotroph, gonadotroph, thyrotroph, and somatotroph; predominant or isolated single-axis dysfunction, such as isolated thyrotroph impairment, is rare [1, 3].

Although LYH is generally considered a localized pituitary inflammatory disorder, cases with cerebrospinal fluid (CSF) pleocytosis and aseptic meningitis have been reported, occasionally mimicking bacterial meningitis [4, 5]. Extension of inflammation from the pituitary to the meninges is uncommon.

Herein, we report a rare case of suspected LYH complicated by meningitis due to direct extension of pituitary inflammation, with reversible predominant thyrotroph dysfunction despite preserved corticotroph function.

## Case presentation

A 65-year-old woman with osteoporosis presented with headache onset 63 days before admission. Head computed tomography (CT) at a local clinic 56 days before admission revealed no abnormalities. Fifty days before admission, she developed a fever of 38 °C and was managed symptomatically. Forty-one days before admission, diplopia due to left abducens nerve palsy developed, for which oral prednisolone was prescribed (30 mg/day for 6 days, followed by 15 mg/day for 6 days), resulting in temporary improvement; however, headache persisted. Fourteen days before admission, diplopia recurred, and low-dose prednisolone (5 mg/day) was reintroduced until admission without sustained benefit. Due to persistent headache, diplopia, nausea, and dizziness, she was referred and admitted. On admission, height and weight were 151 cm and 37.2 kg, respectively. Blood pressure was 175/90 mmHg, pulse rate 58 beats/min, and body temperature 37.6 °C. No polyuria or excessive thirst was noted. Neuro-ophthalmologic examination showed left ptosis and left-predominant bilateral abduction deficits (right abduction impaired, left abduction absent), with mild limitation of left adduction and upward gaze; convergence was also impaired. No visual field defect, limb weakness, sensory deficit, or cerebellar signs were present.

## Diagnostic assessment

General laboratory testing on admission demonstrated neutrophil-predominant leukocytosis and a marked increase in C-reactive protein, indicating severe systemic inflammation (Table 1). Despite markedly elevated inflammatory markers, procalcitonin was only mildly elevated (Table 1). Chest-to-pelvis CT revealed no bacterial focus, and blood and urine cultures, including mycobacterial cultures, were negative.

**Table 1:** Laboratory and immunological data on admission

Test	Results	Reference range
WBC	**19 390/μL** **(19.39** **×** **10^9^/L)**	3300-8600/μL (3.30-8.60 × 10^9^/L)
Neutrophils	**94.0%**	37-74%
Lymphocytes	**2.3%**	18-53%
Monocytes	3.6%	1-8%
RBC	4.76 × 10^6^/μL (4.76 × 10^12^/L)	3.86-4.92 × 10^6^/μL (3.86-4.92 × 10^12^/L)
Hb	13.8 g/dL (138 g/L)	11.6-14.8 g/dL (116-148 g/L)
Hct	42.1%	35.1-44.4%
Plt	**448** **×** **10^3^/μL** **(448** **×** **10^9^/L)**	158-348 × 10^3^/μL (158-348 × 10^9^/L)
TP	**6.2 g/dL** **(62 g/L)**	6.6-8.1 g/dL (66.0-81.0 g/L)
Albumin	**2.7 g/dL** **(27 g/L)**	4.1-5.1 g/dL (41.0-51.0 g/L)
T-Bil	1.02 mg/dL (17.45 μmol/L)	0.4-1.5 mg/dL (6.84-25.66 μmol/L)
LDH	**223 U/L** **(3.72** **μkat/L)**	124-222 U/L (2.07-3.71 μkat/L)
AST	19 U/L (0.32 μkat/L)	13-30 U/L (0.22-0.50 μkat/L)
ALT	11 U/L (0.18 μkat/L)	7-23 U/L (0.12-0.38 μkat/L)
γ-GT	24 U/L (0.40 μkat/L)	9-32 U/L (0.15-0.53 μkat/L)
ALP	89 U/L (1.49 μkat/L)	38-113 U/L (0.63-1.89 μkat/L)
CK	29 U/L (0.48 μkat/L)	41-153 U/L (0.68-2.56 μkat/L)
Urea nitrogen	11.8 mg/dL (4.21 mmol/L)	8.0-20.0 mg/dL (2.86-7.14 mmol/L)
Creatinine	0.62 mg/dL (54.81 μmol/L)	0.46-0.79 mg/dL (40.66-69.84 μmol/L)
Calcium	9.5 mg/dL (2.38 mmol/L)	8.8-10.1 mg/dL (2.20-2.52 mmol/L)
Sodium	140 mmol/L (140 mmol/L)	138-145 mmol/L (138-145 mmol/L)
Potassium	4.0 mmol/L (4.0 mmol/L)	3.6-4.8 mmol/L (3.6-4.8 mmol/L)
Chloride	**90 mmol/L** **(90 mmol/L)**	101-108 mmol/L (101-108 mmol/L)
HbA1c	**6.1%**	4.9-6.0%
BNP	**109.0 pg/mL** **(109.0 ng/L)**	≤18.4 pg/mL (≤18.4 ng/L)
CRP	**17.35 mg/dL** **(173.5 mg/L)**	<0.14 mg/dL (<1.4 mg/L)
Procalcitonin	**0.32 ng/mL** **(0.32** **μg/L)**	<0.05 ng/mL (<0.05 μg/L)
IgG4	110 mg/dL (1.10 g/L)	11-121 mg/dL (0.11-1.21 g/L)
ACE	9.9 U/L (165 nkat/L)	8.3-21.4 U/L (138.34-356.67 nkat/L)
sIL-2R	413 U/mL (413 U/mL)	121-613 U/mL (121-613 U/mL)
ANA	<1:40	< 1:40
Anti-SS-A Ab	<1.0 U/mL (<1.0 U/mL)	<10 U/mL (<10 U/mL)
MPO-ANCA	<0.2 IU/mL (<0.2 IU/mL)	<3.5 IU/mL (<3.5 IU/mL)
PR3-ANCA	<0.6 IU/mL (<0.6 IU/mL)	<2.0 IU/mL (<2.0 IU/mL)
(1→3)-β-D-glucan	12.0 pg/mL (12.0 ng/L)	≤20.0 pg/mL (≤20.0 ng/L)
T-SPOT	Negative	Negative
Aspergillus Antigen	Negative	Negative

Values outside the reference range are shown in bold.

Abbreviations: ACE, angiotensin-converting enzyme; ALP, alkaline phosphatase; ALT, alanine aminotransferase; ANA, antinuclear antibody; Anti-SS-A Ab, anti-Sjögren syndrome-related antigen A antibody; AST, aspartate aminotransferase; BNP, brain-type natriuretic peptide; CK, creatine kinase; CRP, C-reactive protein; Hb, hemoglobin; HbA1c, hemoglobin A1c; Hct, hematocrit; IgG4, immunoglobulin G4; LDH, lactate dehydrogenase; MPO-ANCA, myeloperoxidase-antineutrophil cytoplasmic antibody; Plt, platelet count; PR3-ANCA, proteinase 3-antineutrophil cytoplasmic antibody; RBC, red blood cell count; sIL-2R, soluble interleukin-2 receptor; T-Bil, total bilirubin; TP, total protein; WBC, white blood cell count; γ-GT, gamma-glutamyl transferase.

**Figure 1:**
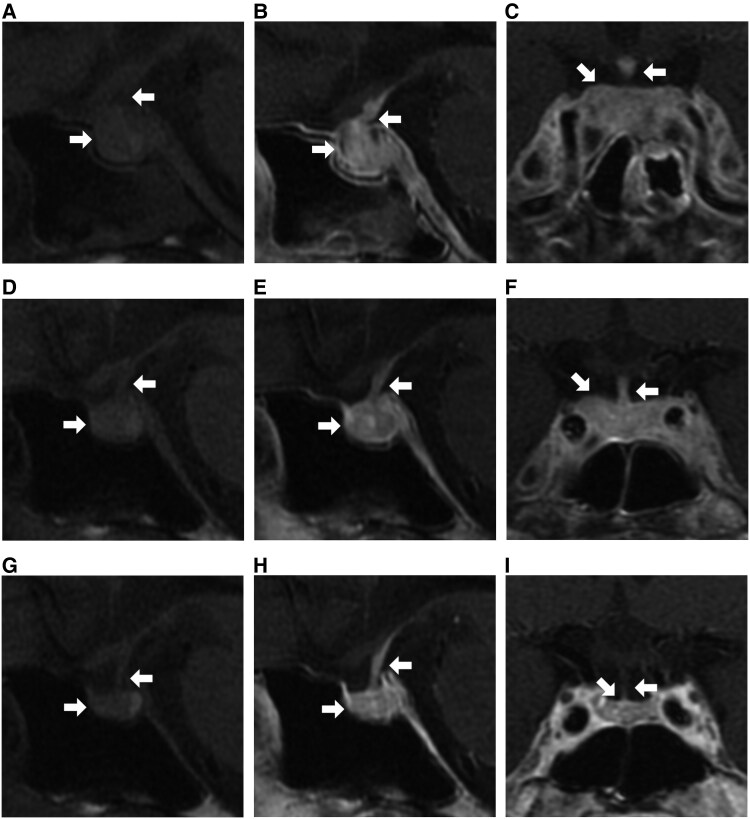
Serial pituitary magnetic resonance imaging (MRI). Noncontrast-enhanced T1-weighted sagittal images (A, D, and G), contrast-enhanced T1-weighted sagittal images (B, E, and H), and contrast-enhanced T1-weighted coronal images (C, F, and I) are shown. On admission (A–C), diffuse enlargement and homogeneous contrast enhancement of the pituitary gland and stalk (white arrow) were observed without optic chiasm compression. On day 21 (D–F), reduction in pituitary enlargement (white arrow) was noted following glucocorticoid therapy. At 5 months after discharge (G–I), further improvement with near-complete resolution of pituitary enlargement (white arrow) was observed.

Head and pituitary magnetic resonance imaging (MRI) performed on admission revealed diffuse enlargement and homogeneous contrast enhancement of the pituitary gland and stalk without optic chiasm compression (Figs. 1A–1C). Although mild inflammatory involvement of the sphenoid sinus was observed on MRI (Fig. 1C), possibly contiguous with mild chronic sinusitis on CT (data not shown), no sinonasal symptoms such as purulent rhinorrhea, nasal congestion, or postnasal drip were noted, and no sellar abscess was identified. These findings were consistent with hypophysitis rather than infectious sinusopathy extending to the pituitary. Lumbar puncture showed colorless, mildly turbid CSF with marked pleocytosis and decreased glucose relative to plasma glucose (Table 2), leading to a diagnosis of meningitis. To exclude autoimmune encephalitis, on day 2, a tissue-based assay (TBA) using rat hippocampus and cerebellum was performed with the patient's serum and CSF. The TBA revealed no abnormal staining, making autoimmune encephalitis unlikely. Cerebrospinal fluid cultures, including mycobacterial cultures, were negative.

**Table 2:** Cerebrospinal fluid analysis

Test	Results	Reference range
Appearance	**Colorless, mildly turbid**	Colorless, clear
Cell count	**1925/μL** **(1925/μL)**	<5/μL (<5/μL)
Mononuclear	**776/μL** **(776** **×** **10^6^/L)**	None
Polymorphonuclear	**1149/μL** **(1149** **×** **10^6^/L)**	None
Protein	**110 mg/dL** **(1.10 g/L)**	10-40 mg/dL (0.10-0.40 g/L)
Glucose	**43 mg/dL** **(2.39 mmol/L)**	50-75 mg/dL (2.77-4.16 mmol/L)
lactate dehydrogenase	**59 U/L** **(0.99** **μkat/L)**	8-50 U/L (0.13-0.83 μkat/L)
Bacterial culture	No growth	No growth
AFB culture	No growth	No growth

Values outside the reference range are shown in bold.

Abbreviation: AFB, acid-fast bacillus.

Endocrinological evaluation on day 2 revealed multiple pituitary hormone abnormalities (Table 3). Free triiodothyronine (FT3) and free thyroxine (FT4) levels were decreased with paradoxically decreased thyroid-stimulating hormone (TSH) levels, suggesting central hypothyroidism (Table 3). Despite being postmenopausal, luteinizing hormone (LH) and follicle-stimulating hormone (FSH) levels were inappropriately low (Table 3). Insulin-like growth factor-1 levels were decreased despite growth hormone (GH) levels remaining within the reference range (Table 3). In contrast, adrenocorticotropic hormone (ACTH) and cortisol levels were elevated (Table 3). Vasopressin secretion was preserved (Table 3), excluding diabetes insipidus. These findings indicated impaired secretion of TSH, LH, FSH, and GH, but not ACTH.

**Table 3:** Serial endocrinological and inflammatory marker data

Test	Day 2	Day 15	Day 22	3 Months after discharge	5 Months after discharge	Reference range
TSH	**0.032** **μIU/mL** **(0.032** **mIU/L)**	0.999 μIU/mL (0.999 mIU/L)	1.016 μIU/mL (1.016 mIU/L)	0.773 μIU/mL (0.773 mIU/L)	0.619 μIU/mL (0.619 mIU/L)	0.610-4.230 μIU/mL (0.610-4.230 mIU/L)
FT3	**1.17 pg/mL** **(1.80 pmol/L)**	**2.26 pg/mL** **(3.47 pmol/L)**	2.69 pg/mL (4.13 pmol/L)	2.95 pg/mL (4.53 pmol/L)	2.92 pg/mL (4.49 pmol/L)	2.52-4.06 pg/mL (3.87-6.24 pmol/L)
FT4	**0.53 ng/dL** **(6.82 pmol/L)**	0.89 ng/dL (11.46 pmol/L)	1.12 ng/dL (14.42 pmol/L)	1.02 ng/dL (13.13 pmol/L)	0.93 ng/dL (11.97 pmol/L)	0.75-1.45 ng/dL (9.65-18.66 pmol/L)
LH	**0.46 mIU/mL** **(0.46 IU/L)**	5.95 mIU/mL (5.95 IU/L)	5.95 mIU/mL (5.95 IU/L)	8.07 mIU/mL (8.07 IU/L)	10.97 mIU/mL (10.97 IU/L)	5.72-64.31 mIU/mL (5.72-64.31 IU/L) (postmenopausal)
FSH	6.49 mIU/mL (6.49 IU/L)	26.79 mIU/mL (26.79 IU/L)	38.81 mIU/mL (38.81 IU/L)	43.09 mIU/mL (43.09 IU/L)	58.16 mIU/mL (58.16 IU/L)	≤157.79 mIU/mL (≤157.79 IU/L) (postmenopausal)
GH	4.11 ng/mL (4.11 μg/L)	0.84 ng/mL (0.84 μg/L)	4.58 ng/mL (4.58 μg/L)	2.09 ng/mL (2.09 μg/L)	6.11 ng/mL (6.11 μg/L)	0.13-9.88 ng/mL (0.13-9.88 μg/L) (Female)
IGF-1	**58 ng/mL** **(7.60 nmol/L)**	92 ng/mL (12.05 nmol/L)	95 ng/mL (12.45 nmol/L)	97 ng/mL (12.71 nmol/L)	111 ng/mL (14.54 nmol/L)	64-188 ng/mL (8.38-24.63 nmol/L) (Female, 65 years old)
PRL	**31.91 ng/mL** **(31.91** **μg/L)**	15.83 ng/mL (15.83 μg/L)	**34.95 ng/mL** **(34.95** **μg/L)**	20.95 ng/mL (20.95 μg/L)	14.32 ng/mL (14.32 μg/L)	6.12-30.54 ng/mL (6.12-30.54 μg/L)
ACTH	**73.0 pg/mL** **(16.06 pmol/L)**	—	—	—	—	7.2-63.3 pg/mL (1.58-13.93 pmol/L)
Cortisol	**44.2** **μg/dL** **(1219.39 nmol/L)**	—	—	—	—	3.7-19.4 μg/dL (102.08-535.21 nmol/L)
DHEA-S	**229** **μg/dL** **(6.18 μmol/L)**	—	—	—	—	12-133 μg/dL (0.32-3.59 μmol/L) (Female, 61-70 years old)
Vasopressin	**3.7 pg/mL** **(3.42 pmol/L)**	—	—	—	—	≤2.8 pg/mL (≤2.58 pmol/L)
Anti-TPO Ab	—	—	<0.50 IU/mL (<0.50 IU/mL)	—	—	<5.61 IU/mL (<5.61 IU/mL)
Anti-Tg Ab	—	—	1.14 IU/mL (1.14 IU/mL)	—	—	<4.11 IU/mL (<4.11 IU/mL)
TRAb	—	—	<0.8 IU/L (<0.8 IU/L)	—	—	<2.0 IU/L (<2.0 IU/L)
Thyroglobulin	—	—	6.91 ng/mL (6.91 μg/L)	—	—	<33.7 ng/mL (<33.7 μg/L)
CRP	**17.35 mg/dL** **(173.5 mg/L)** **(Day 1)**	**0.38 mg/dL** **(3.8 mg/L)**	0.08 mg/dL (0.8 mg/L)	<0.05 mg/dL (<0.5 mg/L)	<0.05 mg/dL (<0.5 mg/L)	<0.14 mg/dL (<1.4 mg/L)
WBC	**19 390/μL** **(19.39** **×** **10^9^/L)** **(Day 1)**	**11 490/μL** **(11.49** **×** **10^9^/L)**	**8830/μL** **(8.83** **×** **10^9^/L)**	6940/μL (6.94 × 10^9^/L)	6550/μL (6.55 × 10^9^/L)	3300-8600/μL (3.30-8.60 × 10^9^/L)

Values outside the reference range are shown in bold.

Abbreviations: —, not performed; ACTH, adrenocorticotropic hormone; Anti-Tg Ab, antithyroglobulin antibody; Anti-TPO Ab, antithyroid peroxidase antibody; CRP, C-reactive protein; DHEA-S, dehydroepiandrosterone sulfate; FSH, follicle-stimulating hormone; FT3, free triiodothyronine; FT4, free thyroxine; GH, growth hormone; IGF-1, insulin-like growth factor-1; LH, luteinizing hormone; PRL, prolactin; TRAb, TSH receptor antibody; TSH, thyroid-stimulating hormone; WBC, white blood cell count.

**Figure 2:**
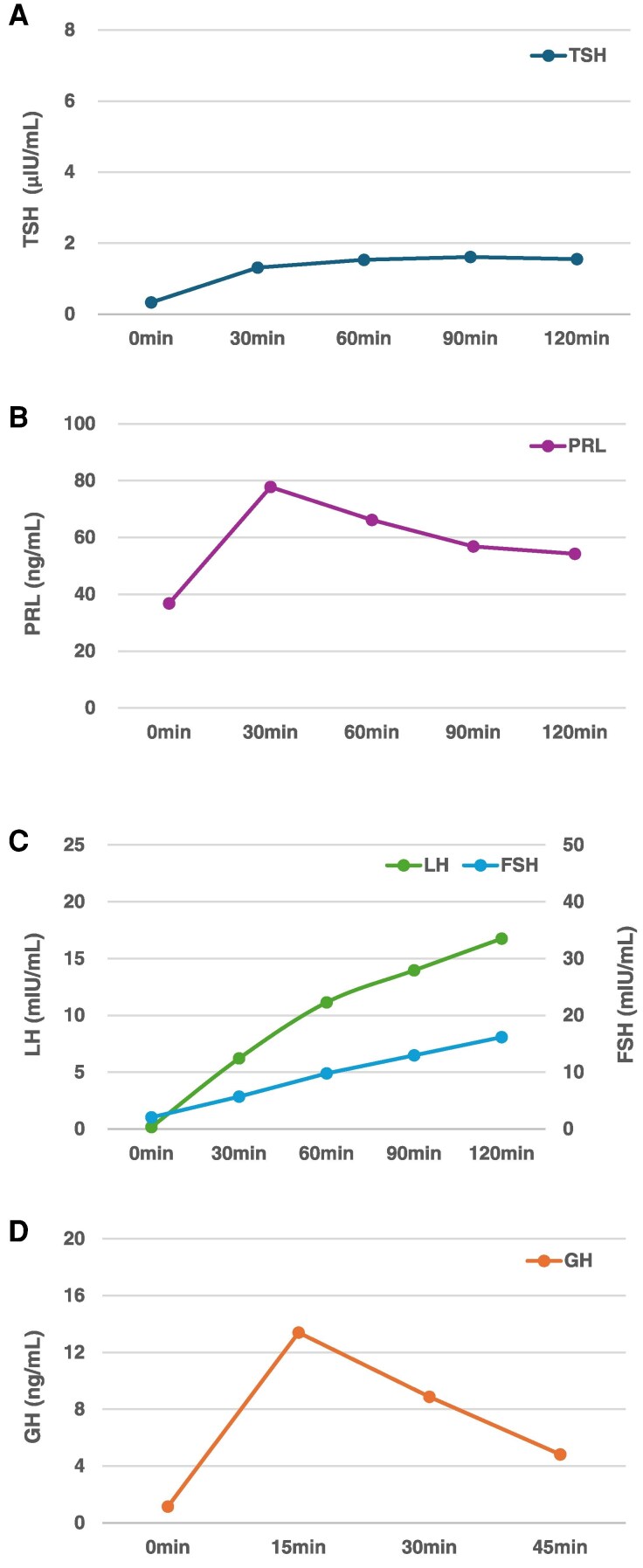
Endocrinological stimulation tests performed before glucocorticoid therapy on days 9 and 10. (A) Thyrotropin-releasing hormone (TRH) stimulation test. Thyroid-stimulating hormone (TSH) levels showed a markedly blunted response to TRH. (B) TRH stimulation test. Prolactin (PRL) levels showed an appropriate response to TRH. (C) Gonadotropin-releasing hormone (GnRH) stimulation test. Luteinizing hormone (LH) and follicle-stimulating hormone (FSH) levels showed appropriate responses to GnRH despite low basal levels. (D) Growth hormone-releasing peptide-2 (GHRP-2) test. Growth hormone (GH) levels showed an appropriate response to GHRP-2, excluding GH deficiency. TSH, PRL, LH, FSH, and GH values can be converted to SI units by multiplying all values by 1.

To evaluate the degree of secretory dysfunction, endocrinological stimulation tests were performed on days 9 and 10. The thyrotropin-releasing hormone (TRH) stimulation test [6] showed baseline TSH levels (0.33 μIU/mL [0.33 mIU/L]) increased to a peak of 1.61 μIU/mL (1.61 mIU/L) at 90 minutes (Fig. 2A), with baseline prolactin (PRL) levels (36.78 ng/mL [36.78 μg/L]) peaking at 77.79 ng/mL (77.79 μg/L) at 30 minutes (Fig. 2B). In healthy individuals, peak TSH levels reach approximately 10 μIU/mL (10 mIU/L) within 15–30 minutes [7], with peak PRL levels exceeding 2-fold baseline or 30 ng/mL (30 μg/L) [8, 9]. Thyroid-stimulating hormone responsiveness was severely impaired, whereas PRL responsiveness was preserved. The gonadotropin-releasing hormone (GnRH) stimulation test [6] showed baseline LH and FSH levels (0.18 and 2.07 mIU/mL [0.18 and 2.07 IU/L], respectively) peaking at 16.76 and 16.19 mIU/mL (16.76 and 16.19 IU/L), respectively, at 120 minutes (Fig. 2C). In healthy adults, peak LH and FSH levels reach 7- to 10-fold and exceed 2-fold increases from baseline, respectively [10]; these results indicated appropriate responsiveness despite low basal gonadotropin levels. The GH-releasing peptide-2 (GHRP-2) test [6] showed GH levels peaking at 13.4 ng/mL (13.4 μg/L) at 15 minutes (Fig. 2D), excluding GH deficiency (threshold: 9 ng/mL [9 μg/L] [11]). Taken together, TSH secretory function was predominantly impaired, likely due to hypophysitis, relative to other anterior pituitary hormones.

Pituitary biopsy is required for the definitive classification and diagnosis of hypophysitis. However, based on clinical, serological, CSF, endocrinological, microbiological, and imaging findings, a presumptive diagnosis of LYH with secondary meningitis was established without pituitary biopsy.

## Treatment

Intravenous dexamethasone 13.2 mg/day was initiated on admission for LYH with secondary meningitis and administered for 3 days. Pending bacterial culture results from blood, urine, and CSF, intravenous meropenem (6 g/day for 3 days, followed by 1 g/day for 10 days) was administered concurrently and discontinued upon confirmation of negative results. On day 3, levothyroxine 25 mcg/day was initiated for central hypothyroidism but discontinued on day 18 following recovery of TSH secretory function. On day 4, intravenous dexamethasone was transitioned to oral prednisolone 25 mg/day, subsequently tapered by 2.5 mg every 2 to 4 weeks based on symptoms, inflammatory markers, and MRI findings.

## Outcome and follow-up

Clinical symptoms including fever, headache, loss of appetite, and general fatigue improved promptly following glucocorticoid therapy. Although brain-type natriuretic peptide levels were elevated on admission (Table 1), they decreased by day 4. C-reactive protein levels gradually decreased, approaching the reference range by day 15 (Table 3). Procalcitonin levels remained below 0.35 ng/mL (0.35 μg/L) throughout the clinical course and decreased to within the reference range by day 15. Bacterial infection was considered unlikely based on negative bacterial cultures, the absence of infectious foci on CT, and the procalcitonin levels described above, as procalcitonin levels in nonbacterial conditions can be mildly elevated but remain below 1.0 ng/mL (1.0 μg/L) [12, 13]. Furthermore, CSF pleocytosis resolved rapidly following glucocorticoid therapy. Impaired pituitary hormones, including TSH, LH, FSH, and GH, also recovered promptly.

**Figure 3:**
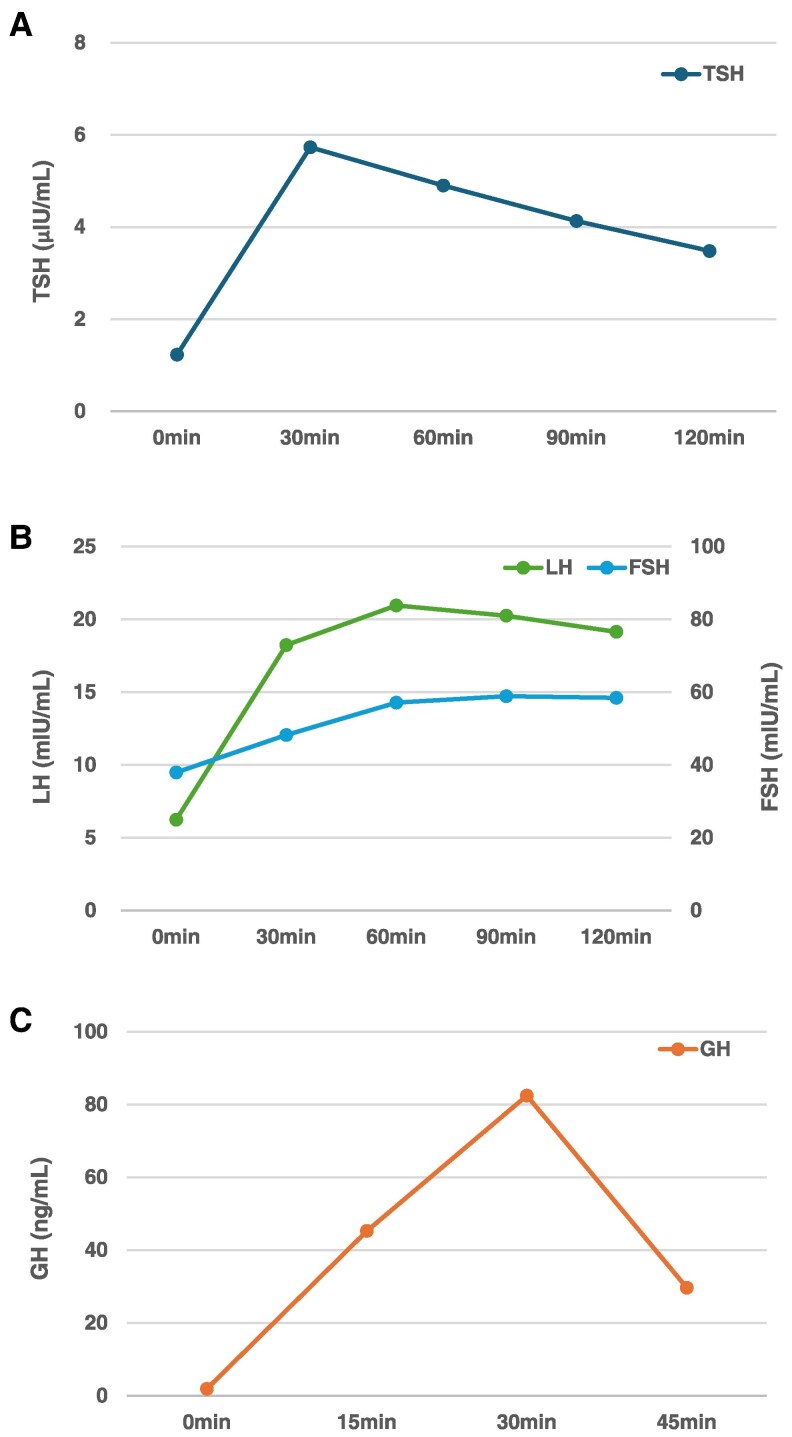
Endocrinological stimulation tests performed on days 29 and 30. (A) Thyrotropin-releasing hormone (TRH) stimulation test. TRH-stimulated thyroid-stimulating hormone (TSH) response showed improved but incomplete recovery. (B) Gonadotropin-releasing hormone (GnRH) stimulation test. Luteinizing hormone (LH) and follicle-stimulating hormone (FSH) levels fell within the postmenopausal range, with an appropriate response to GnRH. (C) Growth hormone-releasing peptide-2 (GHRP-2) test. Growth hormone (GH) levels showed a robust response to GHRP-2. TSH, LH, FSH, and GH values can be converted to SI units by multiplying all values by 1.

Thyroid-stimulating hormone levels improved to 0.999 μIU/mL (0.999 mIU/L) on day 15, with concurrent recovery of FT3 and FT4 levels (2.26 pg/mL [3.48 pmol/L] and 0.89 ng/dL [11.46 pmol/L], respectively) (Table 3), and levothyroxine was discontinued on day 18. A euthyroid state was confirmed on day 22 (TSH 1.016 μIU/mL [1.016 mIU/L], FT3 2.69 pg/mL [4.14 pmol/L], FT4 1.12 ng/dL [14.42 pmol/L]) (Table 3) even after levothyroxine discontinuation. Endocrinological stimulation tests repeated on days 29 and 30 showed baseline TSH levels (1.23 μIU/mL [1.23 mIU/L]) peaking at 5.73 μIU/mL (5.73 mIU/L) at 30 minutes on the TRH stimulation test (Fig. 3A). Baseline LH and FSH levels (6.24 and 37.96 mIU/mL [6.24 and 37.96 IU/L], respectively) fell within the postmenopausal range with appropriate GnRH-stimulated responses (Fig. 3B), and GH levels peaked at 82.5 ng/mL (82.5 μg/L) on the GHRP-2 test (Fig. 3C). These results indicated overall improvement of pituitary secretory function, most notably in TRH-stimulated TSH secretion, although TSH secretory function had not yet completely recovered. As impaired TSH responsiveness to TRH persisted despite resolution of inflammatory findings, whereas other pituitary hormone responses recovered appropriately, nonthyroidal illness syndrome was considered unlikely.

The patient was discharged on day 32 and followed up every 4 to 6 weeks, with prednisolone tapered without complications. Pituitary hormone levels remained within reference ranges throughout the outpatient period, confirming sustained recovery (Table 3). Diplopia and bilateral abduction deficits resolved more slowly than endocrine and inflammatory abnormalities; although abduction deficits persisted initially, ocular motility improved over subsequent months, and the patient resumed driving by 3 months after discharge.

Follow-up MRI on day 21 and at 5 months after discharge showed gradual improvement of pituitary enlargement (Figs. 1D–1I) and prompt resolution of sphenoid sinus inflammation on day 21 (Fig. 1F), consistent with the favorable clinical course.

## Discussion

We report a rare case of suspected LYH presenting with extension of pituitary inflammation to the meninges and reversible predominant thyrotroph dysfunction despite preserved corticotroph function.

Hypophysitis is classified into primary and secondary forms [1-3]. Primary hypophysitis includes autoimmune causes characterized by lymphocytic infiltration (the most common form), IgG4-related disease, and immune checkpoint inhibitor–induced hypophysitis, whereas secondary hypophysitis arises from sellar tumors or cysts, systemic diseases, or infections [1-3]. Primary hypophysitis is further subclassified into histopathological subtypes, including lymphocytic, granulomatous, xanthomatous, IgG4-related, and necrotizing forms [1-3]. Although pituitary biopsy is required for definitive diagnosis, it is reserved for selected cases, and a presumptive diagnosis is typically based on clinical findings, laboratory data, and pituitary MRI [1, 3]. In our patient, the presumptive diagnosis of LYH was based on headache and diplopia, pituitary enlargement with homogeneous contrast enhancement on MRI, anterior pituitary hormone dysfunction, and exclusion of infectious and secondary causes, including IgG4-related hypophysitis. Although serum IgG4 levels were within the reference range at 110 mg/dL (1.10 g/L) (Table 1), below the diagnostic threshold of 135 mg/dL (1.35 g/L) proposed for IgG4-related disease, IgG4-related hypophysitis cannot be entirely excluded without histopathological confirmation.

Lymphocytic hypophysitis often impairs multiple pituitary hormonal axes in descending order of frequency: ACTH, FSH/LH, TSH, and GH; isolated single-axis dysfunction is rare [1, 3]. Adrenocorticotropic hormone deficiency tends to occur earlier in LYH than in other etiologies [1, 3, 14] and may be related to antipituitary or anticorticotroph autoantibodies, though these are not widely available commercially [14]. Kasperlik-Załuska et al [15] reported that patients with secondary ACTH deficiency had a high prevalence of other autoimmune diseases (51%) and positive thyroid autoantibodies (85%), suggesting an association with autoimmune conditions. In contrast, corticotroph function was preserved in our patient, and multiple autoantibodies including thyroid autoantibodies were negative, possibly reflecting the absence of autoimmune mechanisms typically associated with ACTH deficiency. Notably, autoantibody negativity does not exclude LYH, as no autoantibody testing is required for diagnosis [1, 3].

Isolated TSH deficiency in LYH is rare [1, 3]. Barbesino et al [16] reported a link between pituitary autoimmunity and central hypothyroidism, and idiopathic isolated TSH deficiency has been associated with antipituitary autoantibodies in Japan [17]. Lin et al reported that local T lymphocyte activation within the pituitary may induce inflammatory cytokine production, suppressing pituitary cell function [18]. Interleukin-6 (IL-6), a key inflammatory cytokine, acutely suppresses TSH secretion in humans [19]. Given the markedly elevated inflammatory markers in our patient, predominant thyrotroph dysfunction may have been attributable to autoimmune-mediated T lymphocyte activation and IL-6 production. The nonuniform distribution of pituitary cell types [20] suggests that lesion topography may further contribute to the unique pattern of dysfunction, as previously described [1, 3].

High-dose glucocorticoid therapy remains the cornerstone of treatment for LYH [2, 3, 21] and improves endocrinological dysfunction [22]. In our patient, pituitary functions including thyrotroph dysfunction recovered promptly, demonstrating reversibility. Glucocorticoid therapy also contributed to resolution of meningitis from direct extension of pituitary inflammation.

Only a limited number of LYH cases complicated by aseptic meningitis have been reported [4, 5]. Two mechanisms have been proposed [23]: meningitis preceding hypophysitis, with the causative virus triggering antibody formation against the pituitary; or hypophysitis preceding meningitis, with pituitary inflammation extending into the intrathecal space. In our case, the negative CSF culture and the absence of leptomeningeal enhancement on brain MRI, despite diffuse enlargement and homogeneous enhancement of the pituitary gland and stalk, negative TBA results, and rapid resolution of CSF pleocytosis following glucocorticoid therapy collectively support extension of inflammation from the pituitary to the meninges rather than a primary diffuse cerebral inflammatory condition.

In conclusion, we report a rare case of suspected LYH in which pituitary inflammation extended to the meninges, presenting with predominant and reversible thyrotroph axis dysfunction despite preserved corticotroph function.

## Learning points

- Lymphocytic hypophysitis can manifest with an atypical pattern of anterior pituitary hormone dysfunction, including predominant and isolated thyrotroph axis dysfunction.
- Lymphocytic hypophysitis may be complicated by aseptic meningitis due to direct extension of pituitary inflammation to the meninges.
- Early recognition and prompt initiation of glucocorticoid therapy are essential for achieving favorable endocrinological and clinical outcomes.

## Data Availability

Original data generated and analyzed for this case report are included in this published article.
